# Adverse reactions and complications caused by hyaluronic acid injections in the face: A scoping review

**DOI:** 10.1590/0103-644020266888

**Published:** 2026-07-24

**Authors:** Flávia Arpini Ferreto, William Vinícius de Oliveira Santos, Rafael Sarkis-Onofre

**Affiliations:** 1 Graduate Program in Dentistry, Atitus Education -Passo Fundo, Brazil

**Keywords:** Hyaluronic acid, Dermal filler, Complications, Adverse reactions, Facial harmonization, Necrosis

## Abstract

This scoping review identified and mapped the adverse reactions and complications associated with the use of hyaluronic acid (HA) dermal fillers in facial aesthetic procedures. This scoping review was reported according to the PRISMA-ScR. Searches were conducted in three electronic databases (PubMed, Scopus, and Web of Science). Randomized clinical trials, cross-sectional studies, retrospective or prospective cohort studies, case series, or case reports published until 2024 that assessed or reported the use of HA for facial filling in healthy adult patients and documented any adverse reaction or complication were included. Study selection was conducted independently by two researchers. Data extraction was performed on half of the included studies by each reviewer, with cross-checking by a third reviewer. A descriptive analysis of the data was performed, considering the different study designs and the complications and adverse reactions reported. Data were presented in tables and graphs. Fifty-five studies met the eligibility criteria and were included. Most studies were case series and case reports (67.3%). Sample sizes ranged from 1 to 5,000, and participant ages ranged from 18 to 78 years. The most reported adverse reactions among the included studies were bruising (56.4%), ischemia (45.4%), and edema (41.81%), with itching less frequent (3.6%). The most severe complications reported were tissue necrosis (29.1%) and tissue embolism (20%), with some cases leading to blindness or stroke. Bruising and ischemia were the most frequently reported adverse reactions, while tissue necrosis was the most common severe complication reported.

## Resumo

Esta revisão de escopo identificou e mapeou as reações adversas e complicações associadas ao uso de preenchedores dérmicos de ácido hialurônico (AH) na harmonização facial. Esta revisão de escopo foi relatada de acordo com a extensão para revisões de escopo das Diretrizes de Relato Preferenciais para Revisões Sistemáticas e Meta-análises (PRISMA-ScR). As buscas foram realizadas em três bases de dados eletrônicas (PubMed, Scopus e Web of Science). Foram incluídos ensaios clínicos randomizados, estudos transversais, coortes retrospectivas ou prospectivas, séries de casos e relatos de caso publicados até 2024, que avaliaram ou relataram o uso de AH para preenchimento facial em pacientes adultos saudáveis e documentaram qualquer reação adversa ou complicação. A seleção dos estudos foi conduzida de forma independente por dois pesquisadores, cada um extraindo os dados de metade dos estudos incluídos. Foi realizada uma análise descritiva dos dados, considerando os diferentes delineamentos de estudo e as reações adversas e complicações relatadas. Os dados foram apresentados em tabelas e gráficos. Cinquenta e cinco estudos atenderam aos critérios de elegibilidade e foram incluídos. A maioria consistia em séries e relatos de casos (67,3%). O tamanho das amostras variou de 1 a 5.000 participantes, com idades entre 18 e 78 anos. As reações adversas mais relatadas foram equimose (56,4%), isquemia (45,4%) e edema (41,8%), sendo o prurido menos frequente (3,6%). As complicações relatadas mais graves foram necrose tecidual (29,1%) e embolia tecidual (20%), com alguns casos evoluindo para cegueira ou AVC. Conclusão: Equimose e isquemia foram as reações adversas mais frequentemente relatadas, enquanto a necrose tecidual foi a complicação mais comum.

**Figure:**
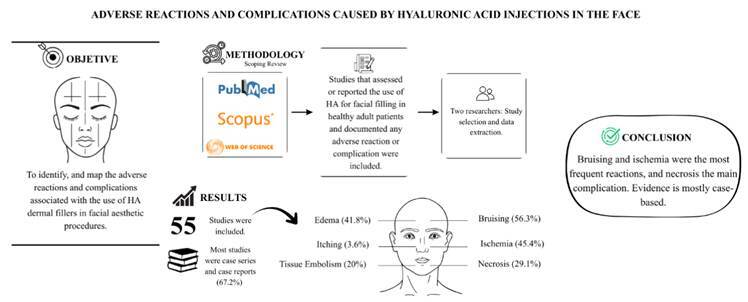

## Introduction

The definition of beauty varies across different cultures. Regardless of age, customs, and gender, beauty is a concern for people^1^. This concern is directly linked to patients' well-being, as aesthetic dysfunction in the facial skin can lead to judgments that hinder self-acceptance^2^. In the human aging process, the face is one of the most visibly affected areas, with the consequences becoming more apparent day by day^3^. This combination drives the search for facial rejuvenation treatments^4^. According to the annual report by the American Society of Plastic Surgeons(2020)^5^, non-invasive facial aesthetic treatments have become increasingly popular in recent years due to their ability to provide natural results that last for a significant period, as their effects are not permanent. Within this context, dermal fillers have emerged as a widely used minimally invasive option due to their proven safety, ease of application, and satisfactory aesthetic outcomes^6^.

One of the most used substances for dermal fillers is hyaluronic acid (HA), a biodegradable and biocompatible product, eliminating the need for skin tests to check for hypersensitivity before the filling procedure^7^. The various HA products available on the market differ in their rheological characteristics (concentration, degree of cross-linking, and viscoelastic properties), allowing for the proper selection of the product for different treatment areas and achieving specific results^8^. Although rare, some adverse reactions associated with HA fillers may occur. However, most of these complications are not severe and tend to resolve as the product is broken down by the enzyme hyaluronidase^6^. In this context, it is crucial that the professionals performing the injections are fully aware of the signs and symptoms related to these complications and are prepared to manage them with confidence.

Although some studies in the literature have addressed adverse reactions and complications related to the use of HA dermal fillers in facial aesthetic procedures, the available evidence remains fragmented across different clinical fields, particularly dermatology and dental/aesthetic practice, with no consolidated interdisciplinary synthesis**.** There is still no comprehensive search that includes, for instance, case reports and case series, which may provide important and practical information for identifying such complications in clinical practice^9^ ^,^ ^10^. Given the heterogeneity of study designs and reported outcomes, as well as the lack of integrated data bridging these disciplines**,** a scoping review is considered the most suitable approach to map and synthesize the complications and adverse reactions associated with HA dermal fillers in facial aesthetic procedures^11^. This approach allows the inclusion and comparison of evidence from different study designs, which is essential in a field where rare but severe complications are reported inconsistently and within discipline-specific literature and across diverse methodological frameworks. Therefore, the aim of this study is to identify and map the adverse reactions and complications associated with the use of HA dermal fillers in facial aesthetic procedures through a scoping review.

## Materials and methods

This study was developed following the recommendations of Peters et al., 2024^11^. The study protocol is available through the link http://osf.io/8uve3. This scoping review was reported according to the Preferred Reporting Items for Systematic Reviews and Meta-Analyses extension for Scoping Reviews (PRISMA-ScR)^12^.

### Eligibility criteria

### Inclusion criteria

The Population, Concept, and Context (PCC) framework was used to define the scope of the review. The Population included healthy adult patients (over 18 years old) who received hyaluronic acid for facial filling. The Concept focused on patients who experienced any type of adverse reaction or complication, and the context was not restricted to any particular setting. Eligible studies included randomized clinical trials, cross-sectional studies, retrospective or prospective cohort studies, case series, and case reports that assessed or reported the use of hyaluronic acid for facial filling in healthy adults and documented any adverse reaction or complication. The adverse reactions considered included edema, erythema, bruising, pain, itching, ischemia, and local inflammatory responses. The complications considered included infections, tissue necrosis, tissue embolism (e.g., blindness or stroke), livedo reticularis, diplopia, and allergic reactions. Nodules were not included, as their etiology is not clearly defined in the literature; some authors associate them with technical errors, the presence of biofilm, or immune system activation. Additionally, the studies were included without language restrictions, and no time restrictions were applied.

### Exclusion Criteria

Studies were excluded if they involved facial fillers other than hyaluronic acid and/or combinations with other substances, pilot studies, cases in which plastic surgery had been performed prior to the filler procedure, or if they focused primarily on injection techniques or device comparisons.

### Information Sources and Search Strategy

The following databases were searched for the selection of studies: PubMed, Scopus, and Web of Science. The search strategy was developed based on PubMed's MeSH terms and adapted for the other databases, and the last search was performed in November 2024. Table 1 presents the detailed search strategy that was used.

**Table 1:** Search Strategy

Database	Search strategy
PubMed	"Hyaluronic Acid"[Mesh] OR "Hyaluronic Acid"[tw] OR “Acid, Hyaluronic” AND "Dermal Fillers"[Mesh] OR "Dermal Fillers"[tw] OR “Fillers, Dermal” OR “Skin Filler” OR “Filler, Skin” OR “Skin Fillers” OR “Fillers, Skin” OR “Dermal Filler” OR “Filler, Dermal” AND "Dermal Fillers/adverse effects"[Mesh] OR “Dermal Fillers/adverse effects”[tw]
Scopus	"Hyaluronic Acid" OR "Acid, Hyaluronic" AND "Dermal Fillers" OR "Fillers, Dermal" OR "Skin Filler" OR "Filler, Skin" OR "Skin Fillers" OR "Fillers, Skin" OR "Dermal Filler" OR "Filler, Dermal" AND "adverse effect" OR "complication" AND ( LIMIT-TO ( DOCTYPE, "ar" ) )
Web of Science	TS=((Hyaluronic Acid OR Acid, Hyaluronic AND Dermal Fillers OR Fillers, Dermal OR Skin Filler OR Filler, Skin OR Skin Fillers OR Fillers, Skin OR Dermal Filler OR Filler, Dermal) AND (adverse effect OR complication)) Refined By: Document type Article

### Selection of sources of evidence

Initially, the search results were transferred to the Rayyan software (Rayyan, Cambridge, MA, USA), where duplicates were removed, and study selection was conducted. A pilot test was performed to assess the agreement between the two reviewers involved in this phase, with references randomly selected using Excel. The two researchers then independently reviewed the titles and abstracts of the articles to assess their relevance and the presence of eligibility criteria. Articles were classified as "include," "exclude," or "undecided." Subsequently, articles classified as "include" and "undecided" were independently selected for full-text reading and further eligibility screening by the same reviewers. In cases of disagreement during the selection of titles/abstracts and full-text studies, the issue was resolved through discussion, and if necessary, a third reviewer was consulted.

### Data charting process and Data items

A standardized data extraction form was developed using Microsoft Excel (Microsoft Excel for Mac). Initially, ten included studies were selected to pilot the data extraction process and ensure consistency in the interpretation of items. The pilot test was followed by a discussion among the three reviewers involved in this phase to resolve discrepancies and refine the process. After evaluating the ten studies selected for the pilot test, inter-rater reliability was assessed using Cohen's kappa coefficient, which showed a value of 0.9. Subsequently, two reviewers (F.A.F. and W.V.O.S.) independently extracted data from half of the included studies each. Additionally, a third reviewer (R.S.O.) verified the consistency of the extracted data.

The following information was collected from each study: authors' names, year of publication, study design, objectives, population/sample, rheology of hyaluronic acid (low, medium, or high), location where the procedure was performed (medical office, dental office, hospital, non-medical sites, other, or not reported), anatomical application site (based on the author's report), amount of hyaluronic acid injected in the region (quantity in mL or not reported), classification regarding the timing of adverse reaction onset (immediate: up to 24h; early: 24h to 30 days; late: after 30 days; not reported), main findings related to complications and adverse reactions, proposed treatment by the author, and conclusions related to the research question. For the purposes of this study, adverse reactions included edema, erythema, bruising, pain, itching, ischemia, and local inflammatory reactions. The complications considered included infections, tissue necrosis, tissue embolism (blindness or stroke), livedo reticularis, diplopia, and allergic reactions.

### Synthesis of results

A descriptive analysis of the data was initially conducted, considering the different study designs included and the complications reported. Categorical data were presented as percentages. To enhance the analysis of information related to anatomical areas, we used the Concept of facial thirds. The upper third extends from the hairline to the eyebrow line; the middle third spans from the eyebrow line to the subnasal line and includes the eyes, nose, cheeks, and ears; and the lower third comprises the region from the subnasal line to the chin. The results were displayed in tables. Additionally, a word cloud was generated to represent the frequency of adverse reactions and complications visually. The ChatGPT platform was used for this purpose. In the word cloud, the size of each term reflects how often it was mentioned across the included studies, highlighting the most frequently reported events.

## Results

**Figure 1:**
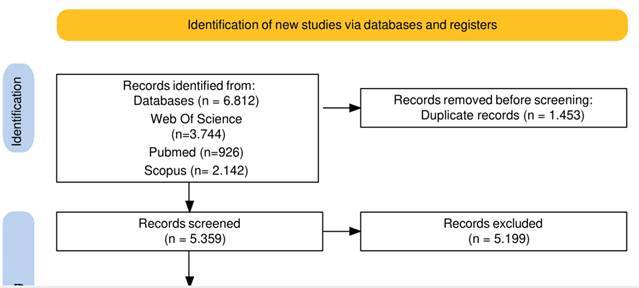
PRISMA flow diagram representing the process of identification of records in databases, duplicate removal, screening, eligibility assessment, and inclusion of studies.

The search in the selected databases resulted in the identification of 6.812 studies. A total of 1.453 duplicates were removed, resulting in 5.359 articles. After analyzing the titles and abstracts, 5.199 were excluded, resulting in 160 remaining articles. Of these, the full text of 27 could not be retrieved. The 133 studies assessed for eligibility had their full texts analyzed, and 78 were excluded (see reasons in the Supplemental Material), resulting in 55 studies included in this scoping review^13^ ^,^ ^14^ ^,^ ^15^ ^,^ ^16^ ^,^ ^17^ ^,^ ^18^ ^,^ ^19^ ^,^ ^20^ ^,^ ^21^ ^,^ ^22^ ^,^ ^23^ ^,^ ^24^ ^,^ ^25^ ^,^ ^26^ ^,^ ^27^ ^,^ ^28^ ^,^ ^29^ ^,^ ^30^ ^,^ ^31^ ^,^ ^32^ ^,^ ^33^ ^,^ ^34^ ^,^ ^35^ ^,^ ^36^ ^,^ ^37^ ^,^ ^38^ ^,^ ^39^ ^,^ ^40^ ^,^ ^41^ ^,^ ^42^ ^,^ ^43^ ^,^ ^44^ ^,^ ^45^ ^,^ ^46^ ^,^ ^47^ ^,^ ^48^ ^,^ ^49^ ^,^ ^50^ ^,^ ^51^ ^,^ ^52^ ^,^ ^53^ ^,^ ^54^ ^,^ ^55^ ^,^ ^56^ ^,^ ^57^ ^,^ ^58^ ^,^ ^59^ ^,^ ^60^ ^,^ ^61^ ^,^ ^62^ ^,^ ^63^ ^,^ ^64^ ^,^ ^65^ ^,^ ^66^ ^,^ ^67^. Figure 1 presents the flow diagram of the study selection process.

The individual characteristics of each study are presented in the supplementary material and organized into three tables:^1^ the characteristics of the included studies related to study design, objectives, Population, and setting (Supplementary Material Table 2);^2^ the characteristics of the included studies related to rheological and procedural details (Supplementary Material Table 3); and^3^ the main findings related to complications and adverse reactions (Supplementary Material Table 4). Among the included studies, the most frequent study design was case series or case report, with 37 studies (67.3%)^14^ ^,^ ^16^ ^,^ ^17^ ^,^ ^18^ ^,^ ^23^ ^,^ ^25^ ^,^ ^27^ ^,^ ^28^ ^,^ ^29^ ^,^ ^30^ ^,^ ^32^ ^,^ ^33^ ^,^ ^34^ ^,^ ^35^ ^,^ ^36^ ^,^ ^37^ ^,^ ^38^ ^,^ ^39^ ^,^ ^40^ ^,^ ^43^ ^,^ ^44^ ^,^ ^45^ ^,^ ^47^ ^,^ ^48^ ^,^ ^49^ ^,^ ^50^ ^,^ ^54^ ^,^ ^55^ ^,^ ^59^ ^,^ ^60^ ^,^ ^61^ ^,^ ^64^ ^,^ ^65^ ^,^ ^66^. The majority aimed to describe adverse complications resulting from hyaluronic acid injection in the face. In relation to participant inclusion in the studies, some studies involved up to 5.000 patients^63^, while others reported cases of only one patient^14^ ^,^ ^16^ ^,^ ^17^ ^,^ ^18^ ^,^ ^21^ ^,^ ^23^ ^,^ ^25^ ^,^ ^29^ ^,^ ^30^ ^,^ ^32^ ^,^ ^34^ ^,^ ^35^ ^,^ ^37^ ^,^ ^40^ ^,^ ^43^ ^,^ ^44^ ^,^ ^45^ ^,^ ^46^ ^,^ ^48^ ^,^ ^49^ ^,^ ^50^ ^,^ ^55^ ^,^ ^59^ ^,^ ^64^ ^,^ ^65^ ^,^ ^66^ with ages ranging from 18^63^ to 78 years^31^.

As for the rheology of the hyaluronic acid used, only 14 studies provided this information (25.4%)^13^ ^,^ ^22^ ^,^ ^24^ ^,^ ^26^ ^,^ ^28^ ^,^ ^29^ ^,^ ^31^ ^,^ ^34^ ^,^ ^36^ ^,^ ^38^ ^,^ ^42^ ^,^ ^53^ ^,^ ^66^, classifying it as high (n=5, 9.1%)^13^ ^,^ ^26^ ^,^ ^29^ ^,^ ^53^ ^,^ ^66^, medium (n=7, 12.7%)^24^ ^,^ ^30^ ^,^ ^31^ ^,^ ^34^ ^,^ ^36^ ^,^ ^38^ ^,^ ^42^, and low (n=2, 3.6%)^22^ ^,^ ^28^. Regarding the location where the procedures were performed, the majority of the studies did not report (34.5%)^16^ ^,^ ^17^ ^,^ ^18^ ^,^ ^19^ ^,^ ^20^ ^,^ ^21^ ^,^ ^25^ ^,^ ^27^ ^,^ ^43^ ^,^ ^44^ ^,^ ^45^ ^,^ ^46^ ^,^ ^47^ ^,^ ^48^ ^,^ ^49^ ^,^ ^53^ ^,^ ^59^ ^,^ ^60^ ^,^ ^66^, while 18 studies were conducted in medical offices (32.7%)^15^ ^,^ ^24^ ^,^ ^26^ ^,^ ^28^ ^,^ ^30^ ^,^ ^31^ ^,^ ^32^ ^,^ ^33^ ^,^ ^35^ ^,^ ^38^ ^,^ ^40^ ^,^ ^41^ ^,^ ^50^ ^,^ ^52^ ^,^ ^57^ ^,^ ^58^ ^,^ ^63^ ^,^ ^65^, and locations such as non-medical sites (facilities and services unrelated to medicine, doctors, or disease treatment) were reported in 4 studies (7.27%)^14^ ^,^ ^23^ ^,^ ^54^ ^,^ ^61^.

Regarding the classification based on the timing of the adverse reaction, only two studies did not report the reaction time (3.6%)^13^ ^,^ ^46^. Twenty-one studies were classified as immediate reaction (38.2%)^14^ ^,^ ^21^ ^,^ ^23^ ^,^ ^25^ ^,^ ^29^ ^,^ ^30^ ^,^ ^32^ ^,^ ^37^ ^,^ ^40^ ^,^ ^41^ ^,^ ^42^ ^,^ ^43^ ^,^ ^45^ ^,^ ^50^ ^,^ ^51^ ^,^ ^54^ ^,^ ^59^ ^,^ ^61^ ^,^ ^64^ ^,^ ^65^ ^,^ ^67^, eleven studies as late (20%)^15^ ^,^ ^17^ ^,^ ^19^ ^,^ ^20^ ^,^ ^27^ ^,^ ^32^ ^,^ ^35^ ^,^ ^36^ ^,^ ^48^ ^,^ ^49^ ^,^ ^60^, and twenty-one studies as early (38.2%). The amount of hyaluronic acid injected varied between 18 mL(51) and 0.49 mL(52), with an approximate mean of 1.51 mL.

**Figure 2:**
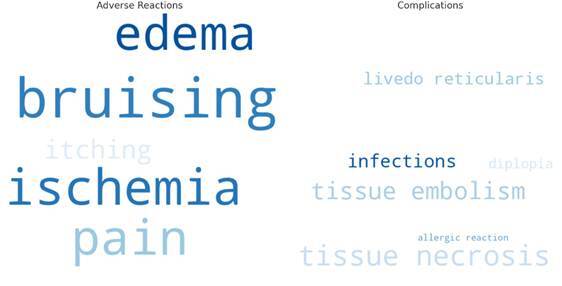
Word cloud illustrating the frequency of adverse reactions and complications reported in the included studies. The size of each term reflects how often it was mentioned, providing a visual summary of the most frequently reported outcomes.

Figure 2 illustrates the word cloud representing the adverse reactions and complications reported in the included studies. The frequency of anatomical filler sites in the included studies was as follows: Midface (65.4%)^15^ ^,^ ^16^ ^,^ ^17^ ^,^ ^18^ ^,^ ^19^ ^,^ ^20^ ^,^ ^21^ ^,^ ^22^ ^,^ ^23^ ^,^ ^26^ ^,^ ^27^ ^,^ ^28^ ^,^ ^30^ ^,^ ^32^ ^,^ ^33^ ^,^ ^34^ ^,^ ^36^ ^,^ ^37^ ^,^ ^38^ ^,^ ^39^ ^,^ ^41^ ^,^ ^44^ ^,^ ^46^ ^,^ ^47^ ^,^ ^50^ ^,^ ^51^ ^,^ ^52^ ^,^ ^53^ ^,^ ^56^ ^,^ ^57^ ^,^ ^58^ ^,^ ^60^ ^,^ ^62^ ^,^ ^63^ ^,^ ^65^ ^,^ ^66^, followed by the Lowerface (49.1%)^13^ ^,^ ^15^ ^,^ ^18^ ^,^ ^19^ ^,^ ^20^ ^,^ ^22^ ^,^ ^24^ ^,^ ^26^ ^,^ ^28^ ^,^ ^29^ ^,^ ^31^ ^,^ ^33^ ^,^ ^36^ ^,^ ^39^ ^,^ ^41^ ^,^ ^42^ ^,^ ^43^ ^,^ ^45^ ^,^ ^47^ ^,^ ^48^ ^,^ ^51^ ^,^ ^56^ ^,^ ^57^ ^,^ ^58^ ^,^ ^61^ ^,^ ^62^ ^,^ ^67^ and finally, the Upperface (27.3%)^14^ ^,^ ^28^ ^,^ ^35^ ^,^ ^36^ ^,^ ^39^ ^,^ ^40^ ^,^ ^42^ ^,^ ^49^ ^,^ ^51^ ^,^ ^54^ ^,^ ^55^ ^,^ ^56^ ^,^ ^58^ ^,^ ^59^ ^,^ ^64^.

Among the main adverse reactions identified were bruising^13^ ^,^ ^17^ ^,^ ^19^ ^,^ ^20^ ^,^ ^22^ ^,^ ^24^ ^,^ ^26^ ^,^ ^27^ ^,^ ^28^ ^,^ ^31^ ^,^ ^32^ ^,^ ^33^ ^,^ ^36^ ^,^ ^38^ ^,^ ^39^ ^,^ ^42^ ^,^ ^46^ ^,^ ^47^ ^,^ ^49^ ^,^ ^50^ ^,^ ^51^ ^,^ ^52^ ^,^ ^53^ ^,^ ^54^ ^,^ ^57^ ^,^ ^58^ ^,^ ^60^ ^,^ ^61^ ^,^ ^62^ ^,^ ^63^ ^,^ ^67^, (56.4%) followed by ischemia^15^ ^,^ ^16^ ^,^ ^17^ ^,^ ^20^ ^,^ ^21^ ^,^ ^23^ ^,^ ^24^ ^,^ ^25^ ^,^ ^30^ ^,^ ^32^ ^,^ ^33^ ^,^ ^35^ ^,^ ^37^ ^,^ ^42^ ^,^ ^44^ ^,^ ^45^ ^,^ ^47^ ^,^ ^49^ ^,^ ^50^ ^,^ ^51^ ^,^ ^54^ ^,^ ^56^ ^,^ ^57^ ^,^ ^58^ ^,^ ^61^, (45.45), edema^16^ ^,^ ^19^ ^,^ ^20^ ^,^ ^24^ ^,^ ^26^ ^,^ ^28^ ^,^ ^33^ ^,^ ^36^ ^,^ ^42^ ^,^ ^43^ ^,^ ^44^ ^,^ ^45^ ^,^ ^46^ ^,^ ^47^ ^,^ ^48^ ^,^ ^49^ ^,^ ^52^ ^,^ ^54^ ^,^ ^55^ ^,^ ^60^ ^,^ ^62^ ^,^ ^64^ ^,^ ^67^, (41.8%), and pain^16^ ^,^ ^19^ ^,^ ^20^ ^,^ ^24^ ^,^ ^26^ ^,^ ^28^ ^,^ ^33^ ^,^ ^36^ ^,^ ^42^ ^,^ ^43^ ^,^ ^44^ ^,^ ^5^ ^,^ ^46^ ^,^ ^47^ ^,^ ^48^ ^,^ ^9^ ^,^ ^49^ ^,^ ^52^ ^,^ ^54^ ^,^ ^55^ ^,^ ^60^ ^,^ ^63^ ^,^ ^64^ ^,^ ^65^ ^,^ ^67^, (43.6%). These percentages refer to the frequency with which each event was reported in the included studies. Itching^22^ ^,^ ^37^ was the least frequently reported adverse reaction, with a prevalence of 3.6%.

Furthermore, the most common complications were: tissue necrosis^16^ ^,^ ^18^ ^,^ ^23^ ^,^ ^29^ ^,^ ^33^ ^,^ ^35^ ^,^ ^41^ ^,^ ^43^ ^,^ ^44^ ^,^ ^45^ ^,^ ^51^ ^,^ ^52^ ^,^ ^56^ ^,^ ^57^ ^,^ ^63^ ^,^ ^66^, (29.1%), tissue embolism^14^ ^,^ ^23^ ^,^ ^25^ ^,^ ^40^ ^,^ ^42^ ^,^ ^49^ ^,^ ^50^ ^,^ ^54^ ^,^ ^56^ ^,^ ^58^ ^,^ ^63^ (20%), infections^20^ ^,^ ^39^ ^,^ ^47^ ^,^ ^58^ ^,^ ^60^ ^,^ ^63^, (10.9%), livedo reticularis^21^ ^,^ ^30^ ^,^ ^33^ ^,^ ^34^ ^,^ ^51^, (9.1%), diplopia^18^ ^,^ ^54^ ^,^ ^64^, (5.4%), and allergic reaction^58^, (1.8%). In the complication classified as tissue embolism, one study reported stroke and blindness as a consequence^14^, (1.8%), while eight other studies reported blindness as a consequence^23^ ^,^ ^25^ ^,^ ^40^ ^,^ ^50^ ^,^ ^54^ ^,^ ^56^ ^,^ ^59^ ^,^ ^65^, (14.5%). Cases of alopecia or tenderness (9.1%) were also found^21^ ^,^ ^28^ ^,^ ^31^ ^,^ ^36^ ^,^ ^37^.

When filtering by study design, no significant differences were observed in the adverse reactions reported in randomized clinical trials. Among the seven included studies^13^ ^,^ ^22^ ^,^ ^24^ ^,^ ^31^ ^,^ ^42^ ^,^ ^46^ ^,^ ^67^, the main adverse reactions identified were swelling/edema, bruising, pain, erythema, itching, and hypersensitivity. These manifestations were mostly mild to moderate and resolved spontaneously. Regarding serious complications, only Barata et al.^2013^ ^)(^ ^42^ reported a case of vascular occlusion; the other studies did not report any serious adverse events or vascular compromise.

Among the treatments for complications, the use of the enzyme hyaluronidase was the most utilized by the authors^14^ ^,^ ^15^ ^,^ ^16^ ^,^ ^17^ ^,^ ^20^ ^,^ ^21^ ^,^ ^22^ ^,^ ^29^ ^,^ ^30^ ^,^ ^32^ ^,^ ^33^ ^,^ ^34^ ^,^ ^35^ ^,^ ^36^ ^,^ ^37^ ^,^ ^38^ ^,^ ^41^ ^,^ ^42^ ^,^ ^43^ ^,^ ^47^ ^,^ ^49^ ^,^ ^50^ ^,^ ^51^ ^,^ ^53^ ^,^ ^54^ ^,^ ^56^ ^,^ ^57^ ^,^ ^58^ ^,^ ^62^ ^,^ ^64^ ^,^ ^65^ ^,^ ^66^, as well as the use of corticosteroids^16^ ^,^ ^17^ ^,^ ^18^ ^,18,^ ^20^ ^,^ ^21^ ^,^ ^23^ ^,^ ^25^ ^,^ ^27^ ^,^ ^29^ ^,^ ^30^ ^,^ ^36^ ^,^ ^37^ ^,^ ^38^ ^,^ ^39^ ^,^ ^40^ ^,^ ^42^ ^,^ ^44^ ^,^ ^48^ ^,^ ^49^ ^,^ ^50^ ^,^ ^51^ ^,^ ^54^ ^,^ ^55^ ^,^ ^56^ ^,^ ^58^ ^,^ ^60^ ^,^ ^63^ ^,^ ^64^ ^,^ ^66^ and antibiotic treatment^14^ ^,^ ^15^ ^,^ ^16^ ^,^ ^18^ ^,^ ^19^ ^,^ ^21^ ^,^ ^22^ ^,^ ^23^ ^,^ ^25^ ^,^ ^30^ ^,^ ^34^ ^,^ ^35^ ^,^ ^37^ ^,^ ^38^ ^,^ ^40^ ^,^ ^41^ ^,^ ^42^ ^,^ ^44^ ^,^ ^47^ ^,^ ^50^ ^,^ ^51^ ^,^ ^54^ ^,^ ^55^ ^,^ ^56^ ^,^ ^58^ ^,^ ^60^ ^,^ ^63^ ^,^ ^64^ ^,^ ^66^. Some authors suggested the use of hyperbaric chamber therapy^16^ ^,^ ^21^ ^,^ ^30^ ^,^ ^39^ ^,^ ^41^ ^,^ ^51^ ^,^ ^54^ and vigorous massage^16^ ^,^ ^22^ ^,^ ^23^ ^,^ ^25^ ^,^ ^30^ ^,^ ^36^ ^,^ ^38^ ^,^ ^42^ ^,^ ^54^ ^,^ ^63^ ^,^ ^65^. The use of analgesic/anti-inflammatory/pain relief methods was also reported in some studies^14^ ^,^ ^16^ ^,^ ^18^ ^,^ ^21^ ^,^ ^22^ ^,^ ^23^ ^,^ ^26^ ^,^ ^30^ ^,^ ^31^ ^,^ ^34^ ^,^ ^37^ ^,^ ^41^ ^,^ ^43^ ^,^ ^47^ ^,^ ^49^ ^,^ ^50^ ^,^ ^51^ ^,^ ^54^ ^,^ ^56^ ^,^ ^58^ ^,^ ^59^ ^,^ ^60^ ^,^ ^63^ ^,^ ^64^ ^,^ ^65^. Less frequently, authors suggested the use of vasodilator medications^21^ ^,^ ^25^ ^,^ ^45^ ^,^ ^51^ ^,^ ^54^, antihistamines^21^ ^,^ ^36^ ^,^ ^39^ ^,^ ^58^ ^,^ ^64^. Some studies did not specify the treatment used^13^ ^,^ ^24^ ^,^ ^28^ ^,^ ^46^ ^,^ ^52^ ^,^ ^67^. Treatments such as topical eye drops^25^ ^,^ ^65^, filler removal with incisions/aspiration^29^ ^,^ ^40^ ^,^ ^47^, epidermal growth factor^37^ ^,^ ^39^ ^,^ ^50^ ^,^ ^54^ ^,^ ^61^, laser irradiation^33^ ^,^ ^39^ ^,^ ^41^ ^,^ ^62^, and the use of a medication known as Minoxidil to aid hair growth^21^ were also suggested. Necrosectomy^18^ ^,^ ^45^, the use of Platelet-rich plasma (PRP)^16^, and finally, recombinant bovine basic fibroblast growth factor gel^35^.

Most conclusions indicate that adverse effects were resolved within days or weeks, especially with the use of hyaluronidase, antibiotics, or corticosteroids. Some reports mention that vision loss due to embolism from hyaluronic acid fillers is often irreversible despite aggressive treatments. Cases of necrosis and ischemia are reported, highlighting the importance of early treatment with hyaluronidase to prevent permanent damage. Some studies report that adverse events were generally mild and transient, resolving spontaneously.

Of the 55 studies, one did not present an outcome^13^. Vision loss was reported in five studies^14^ ^,^ ^23^ ^,^ ^56^ ^,^ ^59^ ^,^ ^65^, with one study reporting blindness in 27 patients^56^. Some studies reported irreversible damage, such as scarring, skin discoloration, and necrosis^16^ ^,^ ^25^ ^,^ ^29^ ^,^ ^35^ ^,^ ^39^ ^,^ ^49^ ^,^ ^54^ ^,^ ^56^ ^,^ ^61^ ^,^ ^64^ ^,^ ^65^. The remaining studies reported complete resolution of adverse reactions and/or complications as the outcome.

## Discussion

This study provides a comprehensive overview of adverse reactions and complications associated with HA dermal fillers in facial aesthetic procedures. Our findings revealed that the main adverse reactions are bruising and ischemia, and the most frequent complication is tissue necrosis. However, it is important to highlight that the evidence is primarily derived from case series and case reports, which, in these situations, serve their role of guiding clinicians in recognizing and managing adverse effects-especially those that are severe.

Although dermal fillers are widely considered safe procedures, our study confirms the occurrence of adverse reactions, in agreement with existing literature. Stefura et al.^2021^ reported predominantly mild and reversible reactions, such as swelling (34%), bruising (29%), pain (28%), and redness (26%)^68^, while Artzi et al.(2016) identified injection-site pain as the most common complaint following the use of monophasic and biphasic fillers^15^. In contrast, our study showed a higher prevalence of bruising (56.4%) and ischemia (45.4%), followed by edema (41.8%), pain (43.6%), erythema (16.4%), and pruritus (3.6%). Similar to recent findings, our results are consistent with the analyses by Kyriazidis et al.(2024)^10^ and Huang et al.(2020)^69^, who also highlighted these as the most frequently reported adverse reactions. These complications are generally short-lived and tend to resolve spontaneously, typically within 14 days.

Regarding the location of adverse reactions, Rayess et al.(2018) reported the cheek (43%) and lip (30%) as the most frequently affected regions^70^, which aligns with our data showing the highest incidence in the midface (65.4%) and lower face (49.1%). Specifically in the midface, more severe adverse reactions have been associated with the periorbital region. This area is commonly treated, as it is one of the first to exhibit signs of facial aging^71^; however, it is also linked to short-term complications, as demonstrated by Mustak et al.(2017)^72^. An analysis of the 13 studies that reported sequelae^14^ ^,^ ^16^ ^,^ ^23^ ^,^ ^25^ ^,^ ^29^ ^,^ ^35^ ^,^ ^39^ ^,^ ^49^ ^,^ ^54^ ^,^ ^56^ ^,^ ^59^ ^,^ ^61^ ^,^ ^65^ revealed that the upper face was the most frequently involved region (7 studies:^14^ ^,^ ^35^ ^,^ ^39^ ^,^ ^49^ ^,^ ^54^ ^,^ ^56^ ^,^ ^59^, followed by the midface (6 studies:^16^ ^,^ ^23^ ^,^ ^25^ ^,^ ^39^ ^,^ ^56^ ^,^ ^65^ and the lower face (3 studies:^29^ ^,^ ^56^ ^,^ ^61^, possibly due to the anatomical and vascular characteristics of these areas. In this context, Park et al.^(^2011)^73^ identified the glabellar region as a high-risk area for necrosis due to potential compression or intra-arterial injection into the supratrochlear artery and also noted the vulnerability of the nasal ala (midface), where limited collateral circulation may be insufficient to compensate for angular artery occlusion.

The rheology of hyaluronic acid (HA) involves parameters such as elasticity (G′), viscosity, and cohesivity^74^. While there is no single product capable of meeting all anatomical and functional demands, understanding the rheological properties of each filler is essential for selecting the most appropriate material for a specific aesthetic or functional goal^75^. Given that different areas of the face present variations in skin tension, muscle activity, and fat distribution, fillers may behave differently depending on the injection site and technique^76^. Our findings indicate that 74.5% of the included studies did not report information regarding the rheological properties of the HA fillers used. This lack of data limits the ability to explore potential correlations between adverse reactions, complications, anatomical regions affected, and the rheological characteristics of the products^77^. Recent studies by Guo et al.(2023)^78^ and Perera et al.(2024)^79^ emphasize that gels with high G′ are more difficult to inject, may cause greater tissue trauma, and are associated with adverse effects such as pain, edema, inflammation, and nodule formation-particularly when there is an imbalance between viscosity and elasticity. In our analysis, it was not possible to assess the incidence of such events, as most of the included studies did not report the specific type of filler used. Nevertheless, these findings reinforce the importance of rheological behavior in predicting outcomes and understanding potential adverse reactions and complications associated with HA-based facial fillers. Standardized reporting of rheological properties in future research would allow for more meaningful comparisons and could help optimize both safety and efficacy in clinical practice. Among the findings related to the locations where the procedures were performed, medical offices were the most frequently reported (32.7%). However, when analyzing the studies with unfavorable outcomes, non-medical sites (facilities and services not related to medicine, physicians, or disease treatment) predominated (30.7%), while 30.7% of the studies did not report information regarding the procedure location. We recognize the importance of biosafety measures and proper training for professionals performing procedures such as facial fillers. Given that our results highlighted the occurrence of adverse reactions and complications, professionals should be adequately prepared to manage these events.

Regarding the onset of adverse reactions and complications, 38.2% occurred immediately (within 24 hours), 38.2% were classified as early reactions (24 hours to 30 days), and 20% as late reactions (after 30 days), while 3.6% of the studies did not report this information. When analyzing the 13 studies that described unresolved complications resulting in permanent sequelae, 61.53% of the adverse reactions and complications occurred immediately, 15.4% were late, and 23.1% were early. These findings are consistent with the study by Almeida et al.(2017), which reports that immediate adverse reactions and complications include vascular changes such as embolization, arterial occlusion, and vision loss^80^. Additionally, our results align with those of Croco et al.(2012), who reported allergic reactions in only 0.1% of cases^81^, which is in line with our study, where only one article^58^ (1.96%) reported an allergic reaction.

This scoping review presents several limitations that warrant cautious interpretation of its findings. The search was conducted in only three databases. In addition, most of the included studies were case reports or case series, presenting methodological heterogeneity and a lack of long-term follow-up data. These aspects lower the overall level of evidence and limit the ability to draw generalizable conclusions. Another limitation is that data extraction was not performed in duplicate. To ensure consistency, however, we conducted a pilot test to standardize the extraction process, and a third reviewer subsequently assessed concordance and verified all extracted data. Finally, this review did not assess differences in adverse reactions related to specific HA fillers, injection techniques, needle size, filler volume, needle versus cannula use, or clinical experience-factors that may influence both the incidence and severity of complications. We recommend that future studies incorporate these variables into their analyses and highlight this gap as an important limitation in the current body of evidence.

Although case reports and case series have small sample sizes and lack control groups-limiting the generalizability of their findings-they can still provide valuable insights into specific adverse events and rare complications associated with HA injections that are often not captured in larger studies. Nonetheless, we strongly recommend conducting studies with greater methodological rigor. Long-term cohort studies may be particularly appropriate for accurately assessing the occurrence of complications and adverse reactions related to HA use. Additionally, in the context of randomized clinical trials, we suggest that extended follow-up periods be implemented to improve the identification and monitoring of these events.

## Conclusion

Our findings indicate that bruising and ischemia were the most frequently reported adverse reactions, while tissue necrosis was the most commonly reported severe complication. Also, indicate that the evidence on adverse reactions and complications associated with hyaluronic acid injections in aesthetic and facial procedures is predominantly composed of case series and case reports, with considerable heterogeneity in methodologies and results. These findings support the need for long-term studies to better understand adverse reactions and complications associated with hyaluronic acid.

## Data Availability

The research data are available upon request.
